# Supraclavicular Subclavian access for Sapien Transcatheter aortic valve replacement- a novel approach

**DOI:** 10.1186/s13019-018-0706-9

**Published:** 2018-01-30

**Authors:** Thom G. Dahle, Nathaniel J. Castro, Brian M. Stegman, Jacob R. Dutcher, John M. Teskey, Wade T. Schmidt, Daren S. Danielson, Sara J. Dezell, Virginia B. Daniels, Daniel J. Tiede

**Affiliations:** CentraCare Heart & Vascular Center, St Cloud Hospital, 1200 Sixth Ave N, St Cloud, MN 56303-1901 USA

**Keywords:** Supraclavicular, Subclavian, Transcatheter, Aortic, Sapien

## Abstract

**Background:**

Within the trans-subclavian approach, procedural techniques can vary widely, and reported access generally refers to an infraclavicular axillary approach. We describe and report the use of a novel supraclavicular true subclavian approach for transcatheter aortic valve replacement (TAVR) exclusively for implantation of Sapien 3 valves.

**Case presentation:**

We report our first five consecutive patients undergoing TAVR with a Sapien 3 valve using a standardized subclavian approach at a single center. In-hospital and 30-day complications were reported. The use of this approach resulted in successful implantation in 100% of patients in a safe manner with 0% mortality, stroke, and vascular injury during hospitalization and at 30 day follow-up. The in-hospital pacemaker implantation rate was 20%. The average length of stay was 3 days.

**Conclusions:**

TAVR with Sapien implant can be safely performed with a standardized supraclavicular subclavian approach in patients with unfavorable femoral access.

## Background

Transcatheter aortic valve replacement (TAVR) continues to expand rapidly as a less invasive option for treatment of severe aortic stenosis in patients considered high risk or non-operable for surgical aortic valve replacement. As delivery systems have advanced allowing for smaller diameter sheath sizes, alternative non-femoral access sites including transapical (TA), trans-subclavian (TS), transcarotid (TC), transcaval, and direct aortic (DA) have become less common but still necessary.

Retrospective registry studies have demonstrated similar procedure outcomes between transfemoral (TF) and TS access sites and worse outcomes with TA access [1–5]. However, the majority of reported data with TS access has been performed with CoreValve (Medtronic, Minneapolis, MN). Data from the UK TAVI registry reported by Frohlich et al. demonstrated that of 3962 patients who underwent TAVR from 2007 to 2012, only 188 patients underwent subclavian access, 99% of which with CoreValve [4]. The largest study to report TS access with Sapien (Edwards Lifesciences Inc., Irvine, CA) was by Ciuca et al. from three Italian centers in which, of the 874 total TAVR procedures, 60 patients underwent TS access and 24 of which performed with Sapien XT [5].

Within the TS approach, procedural techniques can vary widely. In the above subclavian registries, the described subclavian approach was technically transaxillary given the infraclavicular approach. While infraclavicular axillary access is well known to cardiothoracic surgeons, supraclavicular subclavian access is more well known to vascular surgeons. To our knowledge, we are the first to describe and report the use of a novel supraclavicular true subclavian approach exclusively for implantation of Sapien 3 valves.

## Case presentation

Beginning in August 2015, we report our first five consecutive patients undergoing TAVR with a Sapien 3 valve using a standardized subclavian approach at a single center at St. Cloud Hospital, in St. Cloud, MN. All patients had severe symptomatic aortic stenosis as confirmed by echocardiography and determined to be high-risk for surgical aortic valve replacement by two cardiovascular surgeons. Contrast CT of the chest, abdomen, and pelvis was performed on all patients to screen the size of the iliofemoral and subclavian arteries. Patients were found to have iliofemoral or aortic anatomy that was compromised due to severe tortuosity, calcification, small caliber, bilateral iliofemoral bypass, or abdominal aneurysm. All patients underwent pre-procedure coronary angiography as well as subclavian angiography. The left subclavian artery was used in all cases given more favorable delivery angulation relative to the aortic valve and that the left common carotid usually has a separate takeoff from arch. Prior to this, we had only performed TAVR from a supraclavicular subclavian approach on 7 patients with CoreValve and 5 patients with a Sapien XT valve.

**Fig. 1:**
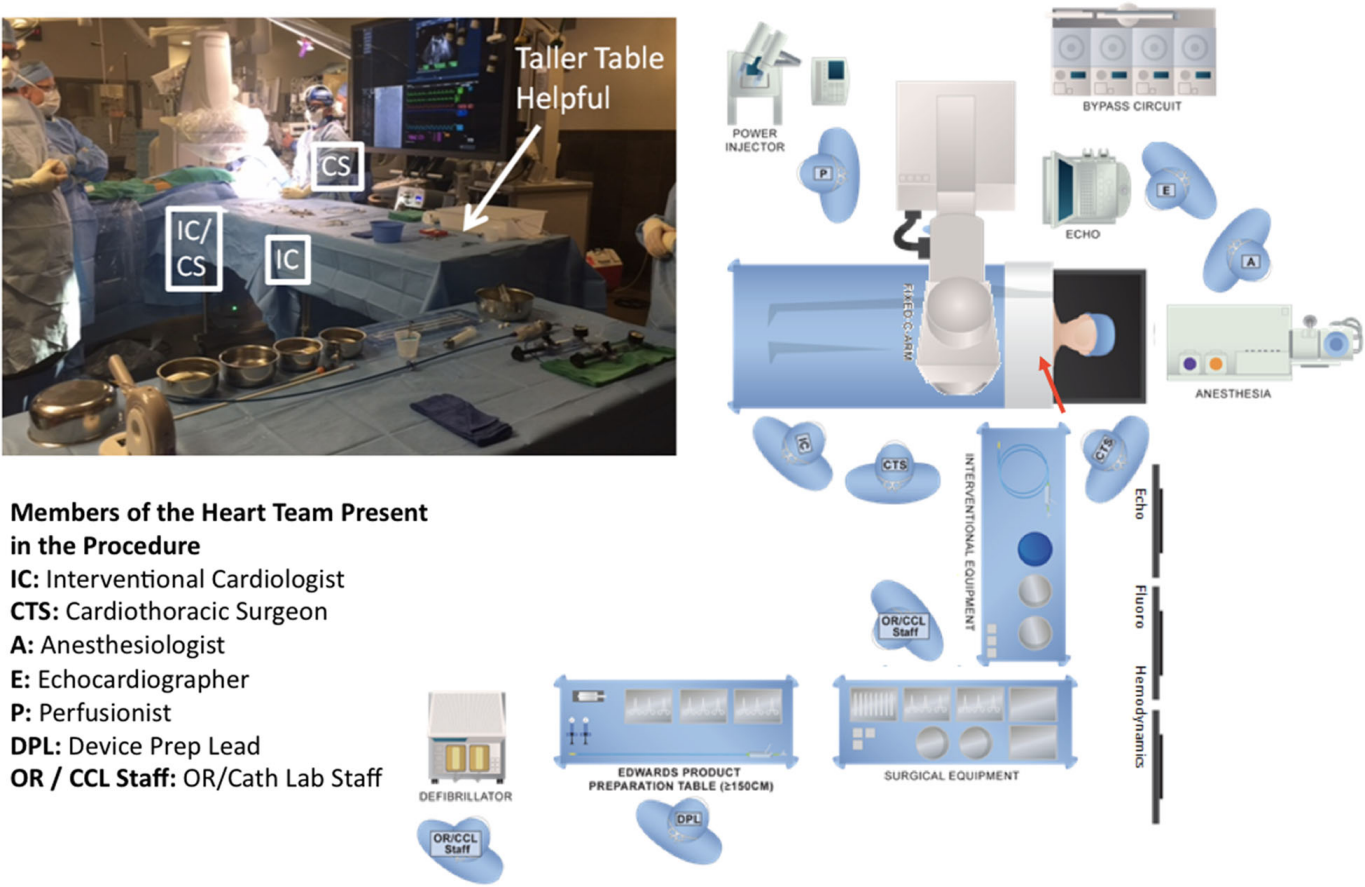
Room setup

**Fig. 2:**
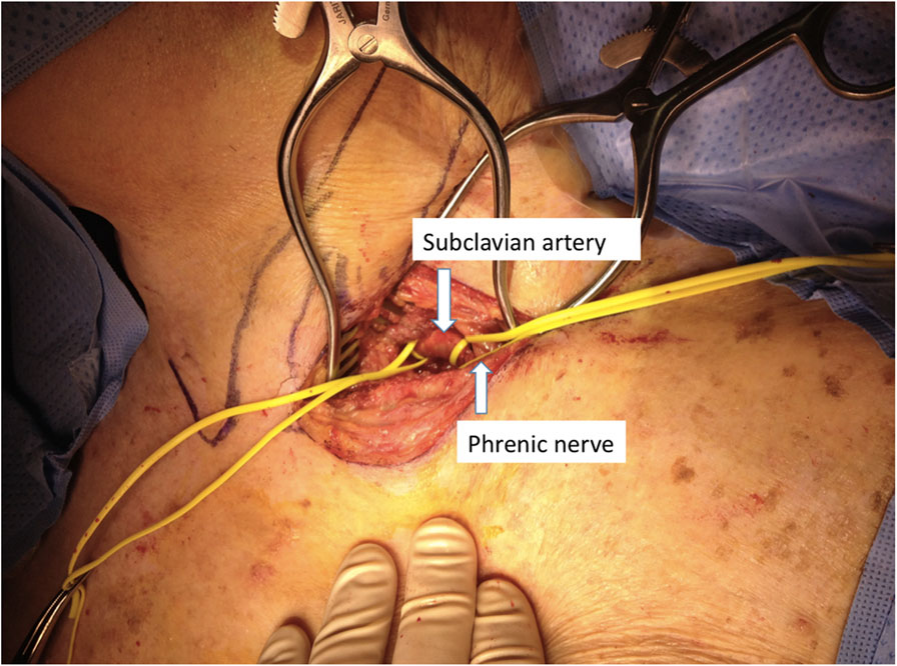
Supraclavicular access with exposed subclavian artery

We determined that tables and monitors are best positioned (as pictured in Fig. 1) off the patients left shoulder to reduce flexion and torque on the valve delivery system. All patients were intubated in a standard fashion. Temporary pacemaker and 6 French (Fr) pigtail were placed via femoral access. A 4–5 cm incision was made superior to the clavicle over the lateral head of the sternocleidomastoid muscle. The lateral head of the sternocleidomastoid muscle was then transected. Next, careful dissection was performed until the phrenic nerve was identified and retracted. Close observation of diaphragmatic movement during cautery can be a helpful clue when dissecting near the phrenic nerve. The anterior scalene muscle was then transected to expose the subclavian artery (Fig. 2). Finally, a purse string suture was made on the anterior surface of the artery around our chosen access site rather than placement of a conduit due to reports of conduit evulsion during axillary approach.

Patients were then given IV heparin at 100 units/kg for a target activated clotting time (ACT) > 250 s. Then, a direct needle puncture of the artery was performed followed by placement of a standard 6Fr 11 cm sheath. We proceeded with crossing the aortic valve through the 6Fr sheath using our standard catheters and straight wire. Next, an Amplatz Super-stiff 0.035 1-cm tip wire (Boston Scientific, Marlborough, MA) with manually shaped distal curve was placed through a 6Fr pigtail catheter. In our experience, less stiff wires, such as Amplatz Extra-stiff 0.035 wire (Cook, Bloomington, IN) or Safari wire (Boston Scientific, Marlborough, MA), do not provide enough support from a subclavian approach. With the stiff wire in the ventricle, the 6Fr sheath was removed and the vessel dilated with the included sheath dilator followed by insertion of the Edwards eSheath (Edwards Lifesciences Inc., Irvine, CA) with the logo towards patient’s feet. This positioned the sheath seam toward the patient’s head, along the greater curvature of the sheath, which reduced buckling and kinking of the sheath. When possible, we upsized to the 16F eSheath, with 23 mm or 26 mm valve implants, or 18F eSheath, with 29 mm valve implants, to reduce required push force. The sheath tip was advanced to mid ascending aorta to protect aorta during valve insertion. In most patients this does require a portion of sheath dilator to cross the aortic valve.

Balloon pre-dilation was optional depending on native valve characteristics. Next, the valve delivery system was advanced into proximal ascending aorta and the sheath withdrawn as needed to expose the valve. The sheath tip was positioned in the aorta 1–2 cm beyond the subclavian artery ostium and not into subclavian artery unless required to provide perfusion to a LIMA graft. In most patients the nose cone of the unaligned valve did cross the aortic valve. Valve alignment was performed in the ascending aorta during which the nose cone would come back across the AV into the aorta.

The delivery catheter was flexed only as needed for alignment with the annulus plane. Generally the catheter did not require any flexion. Positioning and deployment of the Sapien valve was performed identical to a standard TF technique. Additional post dilation was performed only if needed.

**Fig. 3:**
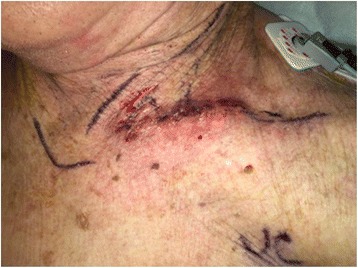
Supraclavicular access site post surgical closure

Following successful valve deployment, confirmed by transesophageal echocardiogram, the valve delivery balloon tip was advanced carefully across the aortic valve and the super stiff wire withdrawn back into the delivery catheter to protect the valve leaflets and subclavian artery from the super-stiff wire. The delivery catheter was then removed. The sheath was then removed and hemostasis achieved by tightening the purse-string sutures. A subclavian angiogram was performed with a standard Judkins right-4 catheter or pigtail from femoral access to evaluate for patency and dissection. Anticoagulation was reversed using protamine sulfate. Surgical closure was performed at the access site (Fig. 3). Placement of a drainage tube was optional and rarely needed.

Post procedure, patients were placed on bed-rest as per protocol for 6Fr groin access only and monitored for vascular or nerve complications. On discharge, patients were instructed to perform no lifting > 10 lbs. with left arm for 4 weeks, no raising of the left arm greater than shoulder height for 1 week, and standard post surgical incision care instructions.

Baseline characteristics of the patients are summarized in Table 1. During this time period, we did not perform TAVR from a transapical access as all patients with non-favorable femoral access were found to have suitable subclavian anatomy.

**Table 1:** Baseline Characteristics of Supraclavicular Subclavian TAVR patients *n* = 5

Age, yrs	85 ± 5.0
Female	3 (30.0)
Height, cm	167.9 ± 5.4
Weight, kg	73.3 ± 13.5
STS PROM	4.7 ± 3.2
NYHA functional class III	5 (100.0)
Prior percutaneous coronary intervention	3 (60.0)
Prior coronary artery bypass grafting	1 (20.0)
Prior myocardial infarction	1 (20.0)
Prior stroke	2 (40.0)
Atrial fibrillation	3 (60.0)
Diabetes Mellitus	0 (0.0)
Hypertension	4 (80.0)
History of tobacco use	3 (60.0)
Chronic kidney disease	2 (40.0)
Peripheral arterial disease prohibiting TAVR	5 (100.0)
Chronic lung disease	3 (60.0)
Porcelain aorta	2 (40.0)
Left ventricular ejection fraction, %	62 ± 8.4
Aortic valve area, mm2	0.80 ± 0.17
Aortic valve mean gradient, mmHg	42.4 ± 1.5

Values are mean SD or n (%)

*NYHA* New York Heart Association, *STS PROM* Society of Thoracic Surgeons predicted risk of mortality

In-hospital mortality was 0%. Procedural success defined as an implanted functioning valve was achieved in all 5 patients (100%). Two patients (40%) required pre-dilation balloon aortic valvuloplasty. No significant procedural complications were seen in any patients including major bleeding or vascular complication. Four patients (80%) had no or trace perivalvular leak post procedure, 1 patients (20%) had mild perivalvular leak post procedure. Average total contrast used was 84 ± 32.7 ml. Average total fluoroscopy time was 14.8 ± 4.1 min.

In-hospital complications included the development of left bundle branch block (LBBB) in 1 patients (20%) and heart block in 1 patient (20%) requiring permanent pacemaker implantation prior to discharge. No patients had minor or major bleeding, stroke, or vascular complication during their hospitalization. Average length of stay was 3.0 ± 1.0 days.

At 30 day clinic follow-up mortality was 0%. 30 day complications were seen in one patients who was admitted with a major gastrointestinal bleed 10 days post procedure.

## Discussion and conclusions

Currently, the only approved non-femoral access with the Sapien 3 valve is via TA and DA access, which has been shown to be associated with worse survival compared to TF access [6].

In this case series, we demonstrate our first 5 consecutive patients who underwent a novel supraclavicular subclavian access approach for implantation of Sapien 3 valves. We demonstrated that use of this approach resulted in successful implantation in 100% of patients in a safe manner with 0% mortality, stroke, or vascular injury during hospitalization and at 30 day follow-up. The in-hospital pacemaker implantation rate was 20% in this small case series. The average length of stay was 3.0 days. In our case series we did not encounter any anatomical limitations with this approach.

Although not directly comparable, the Italian registry of 60 patients who underwent TS access (majority implants with CoreValve) also reported low complication rates with 1% in-hospital mortality, 2% stroke, 3.3% major bleeding, 10% vascular complication, and 27.1% pacemaker implantation (which was driven by valve type with 29.5% patients receiving CoreValve and 7.1% patients receiving Sapien) [5]. Length of stay was not reported.

Data from the UK TAVI Registry of 188 patients who underwent subclavian access (99% receiving CoreValve implant), reported 4.3% in-hospital mortality, 3.0% stroke, 2.0% Major vascular complication, and 23% pacemaker implantation [4]. Average length of stay was 7.0 days (5.0–10.0). This study also demonstrated that in-hospital mortality between the TF group (3.7%) vs SC group (4.3%) was not significantly different (*p* = 0.69); however, mortality in the TA group (9.5%) was significantly higher than the TF group (*p* < 0.0001).

**Fig. 4:**
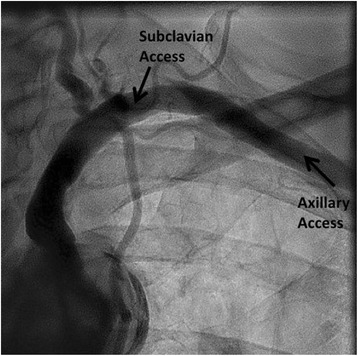
Supraclavicular subclavian vs infraclavicular axillary approach

This case series demonstrated the feasibility of performing TAVR with a Sapien 3 implant using a standardized supraclavicular subclavian approach in patients with unfavorable femoral access. We hypothesize that the advantages of supraclavicular subclavian access over infraclavicular axillary access may include reduced tortuosity due to more direct approach (Fig. 4), easier access in morbidly obese patients, and familiar access for vascular surgeons. Disadvantage of this approach compared to infraclavicular axillary access may include less familiar approach for cardiothoracic surgeons and less suitable for a percutaneous technique. A left subclavian approach is favored over the right subclavian given the more desirable angulation on the left, however a right sided approach could be used if a dependent LIMA graft is present and at risk of occlusion by the sheath (subclavian diameter < 2 mm greater than sheath diameter). We are experienced at both subclavian and axillary approaches and find the supraclavicular subclavian approach very helpful when a percutaneous axillary approach is not favorable due to smaller axillary artery caliber, significant additional tortuosity, or presence of a pacemaker over the axillary access site. However, direct comparison with infraclavicular axillary, TF, TA, and DA approaches needs to be studied with Sapien 3. Currently, we are conducting the ACCESS Study- the first multi-center prospective study evaluating the implantation of the Sapien 3 valve with the above described supraclavicular subclavian approach or an infraclavicular axillary approach compared to TF and TA approaches.

## Abbreviations

ACT: activated clotting time
DA: direct aortic
LBBB: left bundle branch block
LIMA: left internal mammary artery
NYHA: New York Heart Association
STS PROM: Society of Thoracic Surgeons predicted risk of mortality
TA: transapical
TAVR: transcatheter aortic valve replacement
TC: transcarotid
TF: transfemoral
TS: trans-subclavian
